# Histone Modifications in Alzheimer’s Disease

**DOI:** 10.3390/genes14020347

**Published:** 2023-01-29

**Authors:** Dalileia Aparecida Santana, Marilia de Arruda Cardoso Smith, Elizabeth Suchi Chen

**Affiliations:** Department of Morphology & Genetics, Universidade Federal de São Paulo (UNIFESP), São Paulo 04023-062, SP, Brazil

**Keywords:** Alzheimer’s disease, aging, histone modifications

## Abstract

Since Late-onset Alzheimer’s disease (LOAD) derives from a combination of genetic variants and environmental factors, epigenetic modifications have been predicted to play a role in the etiopathology of LOAD. Along with DNA methylation, histone modifications have been proposed as the main epigenetic modifications that contribute to the pathologic mechanisms of LOAD; however, little is known about how these mechanisms contribute to the disease’s onset or progression. In this review, we highlighted the main histone modifications and their functional role, including histone acetylation, histone methylation, and histone phosphorylation, as well as changes in such histone modifications that occur in the aging process and mainly in Alzheimer’s disease (AD). Furthermore, we pointed out the main epigenetic drugs tested for AD treatment, such as those based on histone deacetylase (HDAC) inhibitors. Finally, we remarked on the perspectives around the use of such epigenetics drugs for treating AD.

## 1. Introduction

As life expectancy increases worldwide, there is also an increase in susceptibility to age-associated pathological conditions, especially age-related diseases, such as neurodegenerative diseases [1]. Neurodegenerative diseases are characterized by the aggregation of misfolded neurotoxic proteins, which can start accumulating years before the arising of the first symptoms. These accumulated proteins drive extensive neuronal death and consequent cognitive, motor, or behavioral dysfunction [2].

Alzheimer’s disease (AD) is the most common neurodegenerative disease and the most frequent cause of dementia among the elderly population [3]. The extensive and progressive neuronal loss in the cerebral cortex of AD patients, along with cerebral atrophy and cognitive decline, leads to impaired learning, memory, and daily abilities [4,5]. The hallmarks of AD include the senile plaques—aggregates of β-amyloid (Aβ) peptide in the extracellular space and neurofibrillary tangles—abnormal accumulation of hyperphosphorylated tau protein in neurons [6].

There are two main forms of AD, according to the age of onset. The Early-Onset AD (EOAD) is the autosomal dominant form due to mutations in genes involved with the generation of Aβ peptide: *APP* (amyloid precursor protein gene), *PSEN1* (presenilin 1), or *PSEN2* (presenilin 2) [6,7]. Late-Onset AD (LOAD) is the far most common form of AD. It represents more than 95% of the cases and occurs sporadically. There is no causal gene identified for LOAD, although many previously described variants may increase disease susceptibility. The inheritance of the ε4 allele of the apolipoprotein E gene (*APOE*) was the first identified risk factor associated with LOAD [6,7]. New risk loci have been identified in genome-wide association studies (GWAS) and implicated in different pathways of AD, such as synaptic function (*BIN1*, *CD2AP*, *SORL1*, *EPHA1,* and *PICALM*), cholesterol metabolism (*ABCA7*, *CLU* and *SORL1*), and immune response (*CD33*, *ABCA7*, *MS4A*, *EPHA1*, *CLU* and *CR1*) [8,9,10,11].

New GWAS have been developed with larger sample sizes, and several other risk loci have been identified and associated with AD, including *TMEM106B*, *LILRB2*, *CCDC6*, *TNIP1*, *APP*, *TSPAN14*, *GRN*, *NCK2*, *SHARPIN* [12,13], *CST3*, *USP8, TGFB2* [14], *RABEP1*, *PILRA*, *TP53INP1*, *AP4M1*, *SPI1*, *AP4E1*, *APBB3*, *ZYX* [15], *ACE*, *BCKDK/KAT8*, *ADAM10* [16], *NTN5*, *HAVCR2*, *AGRN* [13], *LRRC25*, *FIBP*, and *KCNN4* [17], as well as enriched pathways such as endocytosis and the activation of microglia and macrophage [12,15,17].

The etiology of LOAD is not completely understood yet. Thus, it has been hypothesized that epigenetic and environmental factors are strongly involved in the development and progression of LOAD through interaction with multiple loci, and all this combined may increase the risk of LOAD [18,19].

Epigenetic modifications have been under the spotlight in the last decades in a wide range of studies concerning complex diseases which cannot be explained merely by genetic variants [18]. The definition of epigenetics comprises the modifications of gene expression in response to environmental stimuli without changing the primary DNA sequence [20]. Epigenetics abnormalities have been widely reported in the onset and progression of several diseases, including AD [21].

Considerable attention has been paid to histone posttranslational modifications (hPTMs). This epigenetic mechanism occurs on the DNA-associated proteins that compose the core histone octamer to form the chromatin structure [19]. Histone posttranslational modifications play an extremely important role in epigenetic regulation by either modulating the chromatin accessibility through their tight bond to DNA or recruiting the binding of other proteins to certain regions of the DNA [22].

Histone modifications have been associated with learning, memory, synaptic plasticity, and cognitive functions, and the dysregulation of these processes was found in mouse models of aging and neurodegenerative diseases, including AD. Thus, efforts have been made to understand the dynamics of histone modifications in AD and how these modifications can be manipulated to develop treatments targeting histone modifications and their associated modifying enzymes in AD pathogenesis [23].

The present review focused on histone modifications and their role in normal and aged brain functions, what has been achieved so far with advances in histone modifications in AD, and the promising possibilities of histone modification-based treatments.

## 2. Histone Modifications in Normal Brain Functions

Histones are important proteins closely bound to DNA, forming the nucleosomes, the basic units of chromatin. Each nucleosome comprises 147 base pairs (bp) of DNA involving a histone octamer, formed by two copies of each canonical histone: H2A, H2B, H3, and H4 [22,24]. H1 histone functions as a linker between two nucleosome units, promoting its stabilization and the high-order packaging of chromatin in the nucleus [25].

Histone N-terminal tails have several sites for posttranslational modifications, such as methylation, acetylation, phosphorylation, ubiquitylation, and SUMOylation, among others [20,24]. These epigenetic modifications to the chromatin structure mediate the accessibility of the genomic information in the nucleus and regulate gene expression in response to a wide range of internal and external conditions [24].

Histone acetylation is the most studied posttranslational modification. It occurs by adding acetyl groups from the cofactor acetyl CoA to amino acid residues of histones N-terminal tails, mostly on lysines (K) of H3 and H4. It is catalyzed by histone acetyltransferases (HATs) and is usually associated with active transcription. In contrast, histone deacetylation is characterized by the removal of the acetyl groups by histone deacetylases (HDACs) [20,26,27] and leads to transcriptional repression [28].

Histone acetylation neutralizes the positive charge of lysines, loosening the interaction between DNA and histones, thus allowing the access of the transcriptional machinery to gene promoters [20,27]. Furthermore, histone acetylation recruits transcription factors (TFs), and facilitates their binding to promoter regions [27,29,30]. However, it is still unclear if the acetylation itself is sufficient to mediate the targeting of the transcriptional machinery [31].

Several mechanisms in the human brain depend on histone acetylation, including memory formation, consolidation, and synaptic plasticity [31]. In the hippocampus, histone acetylation was shown to participate in the formation of long-term memory and excitatory synapses, which in turn is important for the most common forms of synaptic plasticity, including long-term potentiation (LTP) [32].

Studies involving animal models have extensively analyzed the role of histone acetylation in learning, memory, and synaptic plasticity. Lubin et al. reported increased levels of H3 acetylation at the *Bdnf* promoter region in the hippocampus of adult mice after contextual fear conditioning, a condition associated with impaired learning and memory [33]. Moreover, histone acetylation seems to be increased in regions targeted by the transcription factor NF-κB in genes associated with memory consolidation [34,35]. Furthermore, adult mice expressing a mutant form of CREB binding protein (CBP) with suppressed HAT activity showed impaired long-term memory consolidation. Interestingly, the phenotype was re-established upon the reversal of HAT activity or the use of HDAC inhibitors (HDACi) [36]. Additionally, impaired LTP and long-term memory formation were also observed in CBP mutant mice, evidencing the role of histone acetylation in memory and synaptic plasticity [37,38].

**Figure 1:**
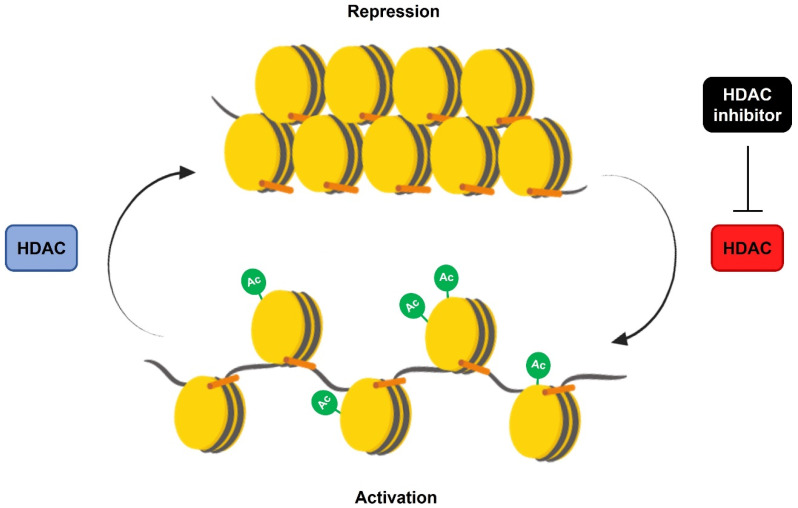
The action of histone deacetylase inhibitors (HDACi) on histone acetylation and chromatin accessibility. HDACs promote the loss of histone acetylation by the removal of acetyl groups, which in turn causes a conformational change in the chromatin structure and, consequently, gene repression. In contrast, the use of HDACi prevents the action of HDACs, maintaining histone acetylation marks and activating gene expression.

Recently, HDACi has been used as a therapeutic intervention, presenting promising results [39]. HDACi increases histone acetylation by targeting HDACs, preventing the deacetylation of histones, and mediates chromatin accessibility, thus leading to active gene expression [40] (Figure 1).

HDACi has been implicated in the improvement of learning, memory, and cognitive impairments [30,41,42,43,44], hence, showing a neuroprotective role [39]. Most HDACi are often referred to as pan-HDACi due to their non-specific inhibitory role, targeting nearly all HDAC classes, while others have an isoform-specific potential [44,45]. However, whether HDACi acts globally or specifically at genes associated with learning and memory has long been in question. A more specific role of HDACi has been demonstrated in the restoration of histone acetylation in mice aged brain after administration of the HDACi suberoylanilide hydroxamic acid (SAHA), specifically in neuronal cells, but not in nonneuronal ones [46]. Vecsey et al. also studied the specificity of an HDACi, trichostatin A (TSA), and reported a specific increase in gene expression during memory consolidation and synaptic plasticity in mice hippocampus. Such increases in gene expression were observed in CREB:CBP-regulated genes, such as *Nr4a1* and *Nr4a2*, which are key genes involved in normal memory formation and LTP [41].

Unlike acetylation, the effect of histone methylation on gene expression can be associated with either activation or repression of gene expression. This transcriptional modulation is dependent on which amino acid residue is methylated and the amount of methyl groups added to each residue. For instance, lysine (K) 9 monomethylation of histone H3 (H3K9me) is associated with transcriptional activation, while di- or trimethylation of the same residue (H3K9me2 or H3K9me3) promotes transcriptional repression; the same pattern is observed for lysine 27 methylation of histone H3 (H3K27) [20].

Histone methylation dynamics is maintained by the opposing actions of histone methyltransferases (HMTs) and histone demethylases (HDMs), which, respectively, methylate and demethylate lysine and arginine residues of histone proteins [47,48]. Moreover, histone methylation recognizes and binds to the chromodomain and other protein domains of chromatin regulators in a protein-protein interaction manner, regulating the transcriptional activity [47,48,49]. Additionally, histone methylation has been reported to act together with DNA methylation in the regulation of gene expression and chromatin remodeling in the brain [50,51,52].

Histone methylation has also been implicated in a number of brain functions, mostly memory formation. The role of histone demethylases has already been related to neurodevelopment, synaptic maturation, and memory processes [53]. In addition, Gupta-Agarwal et al. associated H3K9me2 with transcriptional regulation during memory consolidation in rat hippocampus and entorhinal cortex [54].

Phosphorylation of histone tails is involved in several cellular and molecular mechanisms, such as chromatin remodeling, mitosis, DNA damage repair, and apoptosis [27,55]. Histone phosphorylation is frequently associated with active transcription and, as much as acetylation, promotes the relaxing of the chromatin structure through the neutralization of histone positive charge [27]. This histone modification occurs by the addition of a phosphate group to serines, tyrosines, and threonines by histone kinases, while the removal of the phosphate groups is made by histone phosphatases [27,55]. The role of histone phosphorylation in brain functions has been associated with memory formation pathways [56] and transcriptional regulation of immediate-early genes [57], that have been used as markers of neuronal activity due to their rapid upregulation in response to neuronal and synaptic stimulation [58,59].

Lysine ubiquitylation and SUMOylation are histone modifications far less studied. Nevertheless, these modifications have been implicated in transcriptional regulation, DNA damage response, and signal transduction. They have also been associated with neuroprotection by triggering the clearance of aggregates formed by toxic proteins, such as Aβ. Therefore, they seem to play an important role in neurodegenerative diseases [60,61].

## 3. Histone Modifications in Aging

Aging is characterized by a series of changes that occur in a gradual and time-dependent manner, which promotes a decrease in physiological processes at cellular and molecular levels; thus, it affects immune and metabolic responses while increasing the susceptibility to diseases, such as diabetes, cancer, cardiovascular and neurodegenerative diseases [22,62]. The aging process is accompanied by several mechanisms characterized as the hallmarks of aging, including, but not limited to, genomic instability, changes in epigenetic mechanisms, cellular senescence, loss of protein homeostasis, telomere shortening, and mitochondrial dysfunction [63].

Genetic factors play an extremely important role in age-associated mechanisms, although they are not able to explain all those changes observed in organisms during aging. Studies involving monozygotic twins have highlighted mechanisms responsible for phenotypic differences in the aging processes, i.e., differences that could not be explained by genetic factors. Some differences arise from epigenetic mechanisms, such as DNA methylation and histone modifications [22,64,65].

An important mechanism observed during aging, from yeast to humans, is the loss of heterochromatin, resulting in the loss of transcriptional silencing in certain genomic regions. This process is strongly associated with a shortened life span as it leads to genomic instability [22,66]. Also, the loss of heterochromatin is coupled with the decline of nucleosome occupancy during aging, and they seem to contribute to age-associated genome instability [67,68,69]. Furthermore, diseases associated with premature aging, such as Hutchinson–Gilford progeria syndrome (HGPS) and Werner syndrome, are used as models to study some characteristics of the aging process, and a genome-wide loss of heterochromatin in such diseases has also been reported [70,71].

Global DNA methylation also decreases with aging in almost all mammalian individuals, and it contributes to the observed aging-associated loss of heterochromatin, while some genomic regions are characterized by age-related locus-specific hypermethylation [22,72,73].

Unlike DNA methylation, and considering the great number of existing sites for histone modifications across the genome, it is way more difficult to find patterns of changes in histone modifications in the aging process. In general, there is a decrease in repressive histone marks, along with an increase in activating histone marks with age, characterizing another possible hallmark of aging [74,75]. However, patterns of specific histone modifications can vary across different organisms and even across tissue types [75]. Nonetheless, some specific histone modifications have been reported to have a pattern of age-associated changes. H3K4me3, H4K20me3, and H4K16ac seem to increase with age, while the inverse pattern is observed for H3K9me and H3K27me3, which tend to decrease in aging [62,63], although there is an inconsistency and other distinct patterns have already been reported [68].

Peleg et al. observed failed memory consolidation in the hippocampus in aged mice brains, associated with the deregulation of H4K12ac. When H4K12ac levels were restored, learning and cognitive abilities and the expression of associated genes were recovered [76]. Using HGPS human fibroblasts, Shumaker et al. reported decreased levels of H3K9me3 and H3K27me3 and increased levels of H4K20me3, also a repressive mark [77]. In addition, levels of H3K9/K14 acetylation were reduced at the promoter of genes involved in neurotransmission, signal transduction, GTPase activity, myelination, and mitochondrial function in the aged human prefrontal cortex [78]. Furthermore, Nativio et al. revealed enrichment of H4K16ac in genes associated with neuroplasticity and chromatin modification pathways in aged postmortem samples from the human lateral temporal lobe compared to young and AD subjects [23].

As DNA methylation, a global loss of histone acetylation occurs in the aging process, leading to a transcriptional decline in several genes. Moreover, an exchange of canonical histones for histone variants and the accumulation of histone H3.3 has been reported in aged mouse neurons and the human brain [67,79]. As aforementioned, histone modifications play a crucial role in transcriptional regulation and chromatin remodeling [20,27]. Thus, it is reasonable to further consider the role of the histone-modifying enzymes in aging, not only for the proper functioning of the underlying processes but also for how changes in their dynamics might contribute to the aging process.

The abundance and levels of activity of the histone-modifying enzymes change during aging, but exactly how such changes affect specific pathways and lead to or contribute to aging is not yet completely understood [22].

Mutations affecting both acetylation and deacetylation of histone H3K56 by deletion of the HAT Rtt109 and the HDACs Hst3/Hst4, respectively, lead to a shortened life span in yeast [22,80]. In addition, an extended lifespan was observed upon deletion of genes encoding the catalytic components of the Rpd3 HDAC complex in yeast [22,81], which highlights the complex role of HATs and HDACs on aging.

Aging is also associated with a decline in the expression of sirtuins (SIRT1-7), a family of proteins with HDAC activity [82,83] involved in inflammation, one of the critical mechanisms of aging. By deacetylating the p65 subunit of NF-κB, SIRT1 downregulates a number of inflammation-associated genes, promoting their transcriptional repression [63,84]. Likewise, SIRT2 and SIRT6 are also involved in NF-κB-mediated inflammatory mechanisms [63,85]. Furthermore, an age-related decline in SIRT1 levels was reported in mice microglia, which is thought to exacerbate aging and cognitive decline [86]. Similarly, the role of HMTs in aging was also reported. The inhibition of SUV39H1, an HMT responsible for the trimethylation of H3K9, promoted a decrease in H3K9me3 levels and improved memory and learning in aged mice hippocampus [86].

In summary, conserved patterns of histone modifications have been reported during aging in eukaryotes, ranging from yeast to humans; however, there are also specific patterns across different organisms, tissues, and even cell types. This observation highlights the complexity and the critical role of histone modifications and their related histone-modifying enzymes in several age-related pathways.

## 4. Histone Modifications in Alzheimer’s Disease

Several mechanisms may contribute to the etiopathology of LOAD. It is recognized that the main known genetic risk factor, the ε4 allele of *APOE*, and non-genetic risk factors, such as lifestyle and environmental factors, act together to increase the risk of LOAD [87,88].

Epigenetic mechanisms may modulate gene-environmental interactions and increase the susceptibility or influence on the disease-associated mechanisms [89,90]. Studies in monozygotic twins have been useful in investigating non-genetic factors contributing to the onset of AD, including epigenetic mechanisms and their associated phenotypic differences in monozygotic twins discordant with AD [91].

Alterations in DNA methylation have been associated with age and AD. Differentially methylated regions correlated with the early stages and susceptibility loci for AD [92]. Furthermore, changes in DNA methylation have also been observed in regulatory regions in AD neurons, where it seems to be at an accelerated state compared to normal aging [93].

**Figure 2:**
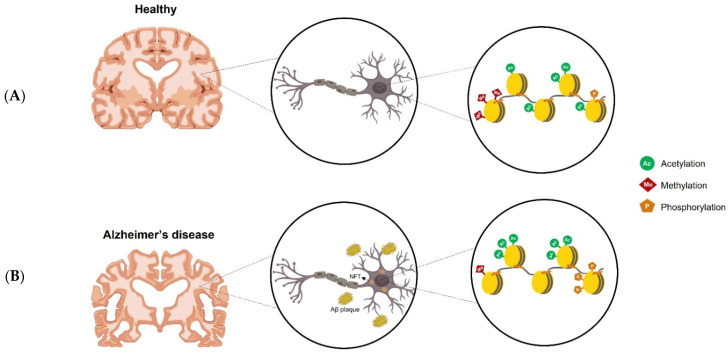
Dysregulation of epigenetic modifications in AD. In the healthy brain, there are a number of epigenetic modifications thar occur on histone tails, such as acetylation, methylation, and phosphorylation (**A**). In AD brains, however, gains or losses of histone modifications can be observed (**B**).

Histone modifications in AD have been evaluated by proteomic, ChIP-qPCR, and other techniques [94,95]. Recently, human large-scale epigenome studies focusing on histone modifications in AD have emerged, helping to elucidate the role of histone modifications on the onset and progression of AD. Furthermore, it has been demonstrated that, while there is a loss of some histone marks in AD, there is also a gain of other marks, demonstrating the complex dynamics of histone modifications in AD [23,96,97,98] (Figure 2).

The main histone modification changes involved in AD pathology will be discussed in details in the following topics, and are summarized in the Table 1.

### 4.1. Histone Acetylation

Histone acetylation plays a role in some crucial mechanisms, such as cognitive functions, memory and learning, response to stress, synaptic plasticity, DNA damage repair, and neuronal death [20,23,30]. Alterations in H3K9ac were correlated with tau-associated pathology and changes in chromatin remodeling in the prefrontal cortex of AD patients compared to elderly controls [96].

In an epigenome-wide analysis of H3K27ac conducted in the entorhinal cortex of AD patients and age-matched low-pathology controls, it was showed that H3K27ac was enriched in genes involved in Aβ and tau pathology, as well as in regions representing LOAD-associated variants [97]. Moreover, Nativio et al. reported decreased H4K16ac across the genome of AD patients compared to controls. These results suggest that AD pathology may be way more complex, presenting distinctive mechanisms from normal aging [23].

By combining transcriptomic, proteomic, and epigenomic analyses, a multi-omics study showed enrichment of H3K27ac and H3K9ac and a loss of H3K122ac in the temporal lobe of AD patients compared to young and elderly controls. These abnormalities were associated with the upregulation of chromatin- and transcription-associated genes, such as *CREBBP*, *EP300*, and *TRRAP*, which encode HATs, including those responsible for the acetylation of H3K9/K27, suggesting a reconfiguration of the epigenome as a mechanism involved in AD pathology [98].

The study of histone acetylation modifiers, such as sirtuins, the class III HDACs, has also provides valuable results for AD research and treatment.

The protein and mRNA expression levels of SIRT6 were found decreased in the brains of AD patients and a FAD mouse model. Mouse hippocampal cells treated with Aβ42 also showed decreased levels of SIRT6, along with increased acetylation levels of H3K9 and H3K56, which are SIRT6 targets. Thus, Aβ42 seems to be implicated in the decrease of SIRT6. Furthermore, SIRT6 overexpression reduced the levels of γH2AX, a marker of DNA damage, and the Aβ42-induced DNA damage. These findings provide more evidence of the role of histone acetylation and its modifier enzymes in AD [109].

Besides SIRT6, SIRT1 has been reported to play an important protective role in AD-associated symptoms. SIRT1 deacetylates histone H1, H2, and H4 residues and other non-histone proteins, such as p53, NF-κB, and RARβ, and is often implicated in anti-inflammatory, antioxidant and anti-apoptotic responses, as well as a role in synaptic plasticity, memory and learning [110]. Overexpression of SIRT1 is thought to reduce the levels of Aβ peptide through increasing α-secretase and, consequently, the preferential activation of the non-amyloidogenic pathway of APP cleavage. It is also reported to prevent the activation of the microglia-mediated release of pro-inflammatory factors due to Aβ toxicity [110].

Animal model studies have also made great advances in understanding the role of histone acetylation in AD. Moreover, the development of HDACi has brought promising results for AD therapeutics.

Inhibition of HDAC3 in an AD mouse model increased histone H3 and H4 acetylation and decreased the accumulation of Aβ and tau phosphorylation while improving learning and memory in such animals [111]. Similar results in Aβ accumulation and tau phosphorylation were also observed in cultured neurons derived from APOE ε4-carrying AD patients [111].

Using a selective inhibitor of HDAC6, Cuadrado-Tejedor et al. observed improved memory impairment and decreased Aβ levels in the hippocampus of the Tg2576 AD mouse model and highlighted the advantages of using a specific HDACi over the pan-HDACi ones [112].

HDACi have been used for the treatment of neurodegenerative diseases for their potential mechanisms in neuroprotection, through the upregulation of neurotrophic factors, in preventing the accumulation of neurotoxic proteins or peptides, such as Aβ, and in the downregulation of pro-inflammatory cytokines [113,114].

The most common HDACi used for the treatment of central nervous system (CNS) diseases include vorinostat (also known as SAHA), valproic acid (VPA), trichostatin A (TSA), and sodium 4-phenylbutyrate (4-PBA), and some of them have been used to treat AD-related symptoms [113,114].

Using VPA, Qing et al. demonstrated that this HDACi was able to reduce the production of Aβ and the formation of senile plaques while improving memory impairment in a transgenic mouse model of AD [115]. In another study, VPA has been shown to enhance neurogenesis through the Wnt pathway and improve learning and memory abilities in the transgenic mice model for AD [116].

Moreover, 4-PBA has also been demonstrated to reverse learning and memory deficits and decrease tau phosphorylation, besides enhancing the transcription of genes involved in synaptic plasticity through increasing histone acetylation levels in a mouse model for AD [117]. In addition to these findings, 4-PBA was also shown to induce Aβ clearance and restore dendritic spine densities in hippocampal neurons [118].

Treatment with the HDACi Suberoylanilide hydroxamic acid (SAHA or vorinostat) in a mouse model of AD has been observed to reverse cognitive deficits and improve memory [119]. However, it was demonstrated that SAHA has a broader distribution on peripheral tissue and a limited effect on the brain [120,121]. Otherwise, the combination of SAHA with other drugs seems to have a synergistic and neuroprotective effect against Aβ and tau pathology and cognitive deficits, and also reduced the levels of oxidative stress and neuroinflammatory markers while increasing the levels of CREB and neurotrophic factors, such as BDNF and GDNF [122,123].

Finally, TSA similarly had a positive effect in reducing senile plaques and improving memory and learning behaviors in APP/PS1 mice. Such results occurred possibly due to its action towards inhibiting Aβ production or enhancing Aβ clearance [124].

Although HDACi treatments have proved to be helpful for AD-related pathological features in mouse models or in vitro studies, there are currently no efficient established HDACi-based treatments for AD patients. However, clinical trials are being conducted and demonstrate promising results for future treatments for AD, in order to overcome the side effects and toxicity presented by the previously tested drugs [125,126].

### 4.2. Histone Methylation

As well as histone acetylation, histone methylation also plays a role in important physiological mechanisms, such as regulation of transcription, alternative splicing, DNA damage responses, DNA replication, chromatin compaction, genome stability, and in a wide range of disease processes, such as cancer and neurodegenerative diseases [52].

The role of histone methylation in AD is less understood, and studies encompassing this histone modification in AD pathophysiology have recently emerged.

An increase in H3K9me2 levels, a repressive histone modification, in the prefrontal cortex of a familial AD (FAD) mouse model was observed. The expression levels of the genes encoding the HATs that catalyze the dimethylation of H3K9, *Ehmt1*, and *Ehmt2* were also increased, as well as their protein levels. Another mouse model of AD also had increased levels of H3K9me2 in the prefrontal cortex. H3K9me2 levels were also increased in the prefrontal cortex of AD patients, as well as the expression levels of *EHMT1*, but not *EHMT2*. In addition, the increased levels of H3K9me2 in the FAD mouse model were associated with decreased levels of the subunits of AMPA and NMDA glutamate receptors. Upon treatment with EHMT1/2 inhibitors, changes in H3K9me2 and glutamate receptors expression levels were reversed, adding more evidence for epigenetic dysregulation in AD and suggesting a therapeutic strategy targeting histone methylation for AD treatment [101].

Wang et al. also observed an increase of H3K9me2 in cortical and hippocampal neurons of mice subjected to induced hypoxia exposure, a condition that increases Aβ production and deposition. Such increases in H3K9me2 were found in the promoter of neprilysin (*NEP*), a gene that encodes one of the proteins responsible for the degradation of Aβ peptide. Thus, the downregulation of *NEP* is associated with the increase of Aβ. Interestingly, the levels of G9a, an HMT that catalyzes the H3K9me2 histone mark, also increased. Knockdown of G9a was able to partially reverse the increase of H3K9me2 and prevented the decrease of *NEP* [127].

Analyzing the levels of H3K4me3, a gene activation-related histone mark, Cao et al. found increased levels of this histone modification in the prefrontal cortex of both AD patients and a mouse model of tauopathy, as well as the levels of the family of HMTs that catalyze this modification. Those changes were associated with impairment of memory-related behaviors and synaptic functions, and tau hyperphosphorylation, which was recovered in the mouse model upon selective inhibition of H3K4me3 HMTs, contributing to understanding the role of histone methylation in AD pathology and providing more basis for novel treatments of AD and tauopathies [103].

In another study, in which the levels of H3K4me3 were assessed in the CK-p25 mouse model of AD, Gjoneska et al. reported an increase in the peak enrichment of this mark in regions associated with immune response pathways, while decreased levels were observed in regions associated with synaptic and learning functions. Similar enrichment patterns were also observed in the hippocampus of AD patients [102].

Besides identifying changes in histone acetylation marks, Nativio et al. also observed changes in a number of histone methylation marks in AD patients compared with elderly controls, with gains (H4K20me2, H3K4me2, H3K27me3, and H3K79me1) and losses (H3K79me2, H3K36me2, H4K20me3, H3K27me1, and H3K56me1) of marks associated with both gene activation and repression, thus highlighting that histone methylation dynamics may be potentially dysregulated in AD [98].

As demonstrated in studies targeting the reversal of dysregulation of histone methylation marks by interfering in the functional role of HMTs and HDMs [101,127], it has been shown that these histone-modifying enzymes have such an important role in this dynamic process. Thus, taking into account the role of histone methylation in memory-related functions and AD, it is reasonable to consider the maintenance of balancing between HMTs and HDMs levels for the proper functioning not only of memory but a range of processes that, once dysregulated, may trigger or contribute to the progression of AD [128].

If increased levels of the histone methylation-modifying enzymes are able to impair memory and cognitive functions, decreased levels may also impair important functions since these proteins are involved, in addition to memory functions in the transcriptional regulation and chromatin modification pathways [129].

Kerimoglu et al. evaluated the knockdown of the lysine methyltransferases Kmt2a and Kmt2b in hippocampal neurons of mice and observed a decrease in H3K4me3 along with impaired memory functions. However, the knockdown of these two lysine methyltransferases (KMT) impaired different gene expression regulatory pathways: genes associated with the regulation of transcription, mRNA processing, and chromatin binding were affected by the knockdown of Kmt2a and genes involved in Wnt signaling, cytokine activity, angiogenesis, and cell adhesion pathways were perturbed by the knockdown of Kmt2b. Additionally, the changes in H3K4me3 observed in neurons of mice lacking Kmt2a were similar to those found in the CK-p25 mouse model of AD neurodegeneration previously reported [102], including decreased H3K4me3 levels in sets of genes enriched for memory- and synaptic plasticity-related categories [130].

The role of HDM in the neurodegeneration process has also been demonstrated. Upon deletion of LSD1, a histone demethylase that demethylates specifically mono and dimethylation of H3K4 (H3K4me1/2), mutant adult mice had widespread neuronal death in the hippocampus and cortex, as well as learning and memory deficits. In addition, the transcriptional changes observed in these animals were similar to those altered in AD and frontotemporal dementia, and LSD1 is co-localized with aggregates of senile plaques and neurofibrillary tangles in AD. These results suggest a possible role of LSD1 in preventing neuronal death and consequent neurodegeneration and also reveal a mechanism of dysregulation possibly involved in AD and other neurodegenerative diseases [131].

Thus, the important involvement of HMTs and HDMs in the regulation of histone methylation levels is clear. It is crucial in the maintenance of the appropriate levels of histone methylation marks. It is also strongly required for the proper balance between the histone methylation-modifying enzymes because either an increase or decrease in their levels may contribute to disease states, including AD. As well as for histone acetylation dynamics, research involving the administration of drugs to control suitable levels of histone methyltransferases and demethylases is emerging and exhibiting promising results for the therapeutic approach of AD [129].

### 4.3. Histone Phosphorylation

Histone phosphorylation is another histone modification that has been reported to play a role in AD pathology, although few studies still address this modification in AD. This hPTM has been associated with transcriptional regulation, DNA damage repair, apoptosis, and chromatin remodeling [27,55]. However, since it has also been associated with neuroplasticity and memory consolidation processes [32,132], it is reasonable to consider its role in other brain functions and even in neurodegenerative diseases, including AD.

Phosphorylation is also a process required for non-histone proteins, such as TFs. Interestingly, the kinase mitogen- and stress-activated protein kinase-1 (MSK1), in addition to phosphorylate histone H3 residues, also mediates the phosphorylation and consequent activation of CREB, a TF that is a key component of the coactivator complex CREB:CBP with HAT activity and is important for transcriptional activation through histone acetylation [133]. Thus, histone modifications are dynamic processes that can act together and are sometimes dependent on each other in a series of processes [132].

An increase in phosphorylation of serine (S) 47 of histone H4 (H4S47p) was found in cells expressing an APP isoform and in Aβ-treated neurons. Therefore, the authors investigated if the same results were observed in brain samples of mild cognitive impairment (MCI) and AD patients. The results showed a slight increase of H4S47p in MCI and a much more significant increase in AD brain samples, demonstrating an APP and/or Aβ-mediated dysregulation in histone phosphorylation in AD [104].

Phosphorylation has also been seen in the histone variant H2AX in brain samples of AD patients. This variant histone is phosphorylated on Ser-139 in response to DNA damage, such as double-strand break (DSB), to form γH2AX. High levels of γH2AX were found in astrocytes of the hippocampus and cortex of AD patients but not in age-matched controls, highlighting the role of astrocytes and DNA damage responses in AD, along with a better understanding of the role of histone phosphorylation in AD [105].

Ogawa et al. also explored histone phosphorylation levels in the brain of AD patients and age-matched controls. They found an increase in phosphorylation of histone H3, specifically at Ser-10 (H3S10p), in the hippocampal tissue of AD patients. Interestingly, phosphorylated H3 was found in the cytoplasm of vulnerable neurons in AD rather than the nucleus. These findings were related to aberrant mitotic machinery and cell cycle activation, indicating a possible mechanism leading to AD neurodegeneration [106].

In accordance with the other studies previously reported, Rao and colleagues found increased levels of histone phosphorylation in AD brains. This increase was observed in total histone H3 in the frontal cortex of AD patients compared to age-matched controls and was associated with an increase in global DNA methylation [107].

Although there are few studies with a focus on histone phosphorylation and its role in AD pathogenesis, the studies published to date contribute to understanding how aberrant histone phosphorylation can impair neuronal and glial functions in AD and contribute to the accumulation of damage and the progressive neurodegeneration observed in the brains of AD patients.

### 4.4. Other Histone Modifications

Histone modifications such as ubiquitylation, SUMOylation, and other hPTMs are far less studied in AD. Regarding ubiquitylation, histones H2A and H2B can be mono- or polyubiquitylated. However, polyubiquitylation has been associated with histone-histone interactions in the nucleosome, monoubiquitylation has been associated with the indication of DNA damage sites. In line with this, the monoubiquitylation of histone H2A was reported to be induced by DNA damage and can be colocalized with phosphorylation of H2AX at DNA damage sites, where these two histone modifications may be required by the nucleotide excision repair (NER) machinery [134].

In an AD study, the levels of H2B ubiquitylation at Lys-120 (H2BK120ub) increased in the frontal cortex of AD patients [108]. However, the role of ubiquitylation in AD is not restricted to histone proteins. The ubiquitin-proteasome system, responsible for the normal degradation of proteins, seems to be impaired in AD and associated with the accumulation of Aβ and paired helical filaments of hyperphosphorylated tau, drawing attention to the role of ubiquitylation of histone and non-histone proteins in AD [108,135].

The evidence of the role of SUMOylation in AD occurs indirectly on histones, affecting HDAC proteins. SUMOylation of HDAC1 has been found on Lys-444 and Lys-476 and seems to regulate its biological activity [136]. In rat hippocampus, it was demonstrated that Aβ injection leads to an increase in Protein inhibitor of activated STAT1 (PIAS1) expression. PIAS1 is an E3 ligase involved in the SUMOylation of a number of proteins, including HDAC1. Thus, increased Aβ-induced PIAS1 protein expression enhanced HDAC1 SUMOylation. In contrast, inhibition of PIAS1 decreased HDAC1 SUMOylation levels. In addition, HDAC1 SUMOylation rescued learning and memory impairment, reduced amyloid plaques and neuronal death in the hippocampus of APP/PS1 mice, and decreased HDAC1 binding to neprilysin and CREB, suggesting a role for HDAC1 SUMOylation in a possible protective mechanism against Aβ-induced neurodegeneration [60].

The evidence observed to date of these less frequently studied histone modifications draws attention to their participation in AD pathology, not only for the study of each of these modifications alone but especially for their interaction with each other, establishing a better view of the interplay between hPTMs, the so-called histone crosstalk, in AD.

## 5. Conclusions and Perspectives

The etiology of LOAD remains elusive; however, much progress has been made in understanding the genetic bases of LOAD, in which multiple loci are involved, combined with environmental and epigenetic factors implicated in its onset and progression.

Epigenetic factors, in particular, have been extensively studied for their ability to modulate gene-environment interactions and influence gene expression patterns. Of these, hPTMs have gained attention for being very dynamic and diverse. In addition, studies with animal models have made a lot of progress and contributed to elucidating the role of histone modifications in the brain and how changes in the patterns of these modifications can influence the pathological state of AD.

It has been demonstrated that changes in histone acetylation, methylation, phosphorylation, and other hPTMs are present in the brain of AD patients. The main goal now is to elucidate whether these changes are able to cause and initiate the pathological state or if they are altered as a consequence of previous changes resulting from an ongoing pathological state.

Furthermore, histone-modifying enzymes seem to be a promising target for AD treatment. In fact, the use of drugs targeting some of these histone-modifying enzymes, such as HDAC inhibitors, had presented promising results in animal models; nonetheless, achieving the beneficial effects of HDAC inhibitors in improving memory and cognition and decreasing the pathological hallmarks and associated neuroinflammation, while controlling the adverse effects caused by these drugs, is still a major challenge.

Although there is no cure for AD, and it is not possible to reverse the neuronal loss that occurs in the brain of AD patients, some of these drugs are being tested in clinical trials with AD patients. In the near future, they may be used at least to treat the symptoms and possibly slow AD progression.

## Figures and Tables

**Table 1:** Main histone modification changes associated with aging and AD.

**Histone Modifications in Aging**	**Change**	**Model**	**Reference**
Global repressive histone marks	↓	Multiple models	[74,75]
Global activating histone marks	↑	Multiple models	[74,75]
H3K4me3	Change in landscape	Mammals	[62,63,99]
H4K16ac	↑	Human brain	[23]
H4K20me3	↑	Mammals Human fibroblasts	[62,63,77,100]
H3K9me3	↓	Human fibroblasts	[77]
H4K12ac	Deregulation	Aged mice brain	[76]
H3K27me3	↓	*C. elegans* Human fibroblasts	[62,63,77,100]
H3K9ac	↓	Human brain	[78]
H3K14ac	↓	Human brain	[78]
Histone variant H3.3	↑	Mouse neurons Human brain	[67,79]
**Histone modifications in AD**	**Change**	**Model**	**Reference**
H3K9ac	↑	Human brain	[96,98]
H3K27ac	↑	Human brain	[97,98]
H4K16ac	↓	Human brain	[23]
H3K122ac	↓	Human brain	[98]
H3K9me2	↑	Mouse models Human brain	[101]
H3K4me3	↑	Human brain Mouse models	[102,103]
H4K20me2	↑	Human brain	[98]
H3K4me2	↑	Human brain	[98]
H3K27me3	↑	Human brain	[98]
H3K79me1	↑	Human brain	[98]
H3K79me2	↓	Human brain	[98]
H3K36me2	↓	Human brain	[98]
H4K20me3	↓	Human brain	[98]
H3K27me1	↓	Human brain	[98]
H3K56me1	↓	Human brain	[98]
H4S47p	↑	Human brain	[104]
H2AXS139p (γH2AX)	↑	Human brain	[105]
H3S10p	↑	Human brain	[106]
Total H3 phosphorylation	↑	Human brain	[107]
H2BK120ub	↑	Human brain	[108]
